# Efficacy and safety of anamorelin in patients with cancer cachexia: Post‐hoc subgroup analyses of a placebo‐controlled study

**DOI:** 10.1002/cam4.5206

**Published:** 2022-11-16

**Authors:** Koichi Takayama, Toru Takiguchi, Naoyuki Komura, Tateaki Naito

**Affiliations:** ^1^ Department of Pulmonary Medicine, Graduate School of Medical Science Kyoto Prefectural University of Medicine Kyoto Japan; ^2^ Clinical Development Planning Ono Pharmaceutical Co., Ltd. Osaka Japan; ^3^ Division of Thoracic Oncology Shizuoka Cancer Center Shizuoka Japan

**Keywords:** anamorelin, appetite, body weight, cancer cachexia, lean body mass, non‐small cell lung cancer

## Abstract

**Background:**

Cachexia, a disorder associated with anorexia, inflammation, and muscle wasting, is frequent in cancer patients. We performed post‐hoc analyses of the ONO‐7643‐04 study to investigate the efficacy and safety of anamorelin in subgroups of Japanese patients with non‐small cell lung cancer (NSCLC).

**Methods:**

The patients were divided into subgroups by baseline characteristics, including sex, age, body mass index, prior weight loss, performance status (PS), concomitant anticancer therapy, and number of previous chemotherapy regimens. The changes from baseline through to 12 weeks for lean body mass (LBM), body weight, and appetite were calculated. Appetite was evaluated using the quality of life questionnaire for cancer patients treated with anticancer drugs (QOL‐ACD) item 8 score. Responder rates were defined as the maintenance/improvement of LBM (≥0 kg), body weight (≥0 kg), or QOL‐ACD item 8 score (≥0) from baseline to all evaluation time points. Safety was evaluated in patients subgrouped by age and PS.

**Results:**

Anamorelin resulted in greater improvements versus placebo in LBM, body weight, and appetite in most subgroups. Anamorelin was also associated with greater LBM, body weight, and appetite responder rates than placebo in nearly all subgroups. Among anamorelin‐treated patients, adverse drug reactions (ADRs) tended to be more frequent with increasing age (<65 years, 19.2%; ≥65 to <75 years, 45.9%; ≥75 years, 60.0%) and PS score (PS 0–1, 38.4%; PS 2, 60.0%). The frequency of serious ADRs was 2.7% and 0% in the PS 0–1 and PS 2 subgroups, respectively.

**Conclusion:**

This study of NSCLC patients with cancer cachexia revealed consistent improvements in LBM, body weight, and appetite across most subgroups of anamorelin‐treated patients. This study also demonstrated the tolerability of anamorelin regardless of age and PS, with a low incidence of serious ADRs in each subgroup.

## Graphical abstract

We performed post‐hoc analyses of the ONO‐7643‐04 study to investigate the efficacy and safety of anamorelin in subgroups of Japanese patients with non‐small cell lung cancer. This study revealed consistent improvements in lean body mass, body weight, and appetite score using QOL‐ACD item 8 across most subgroups of anamorelin‐treated patients versus placebo. This study also demonstrated the tolerability of anamorelin regardless of age and performance status, with a low incidence of serious adverse drug reactions in each subgroup.

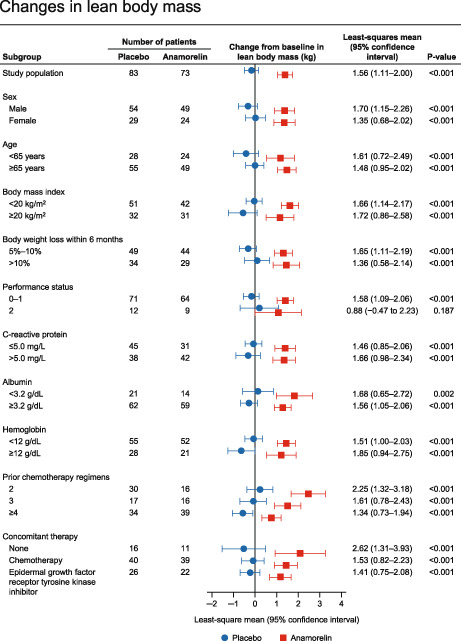

## 1 INTRODUCTION

Cachexia is a disorder associated with anorexia, inflammation, and muscle wasting that frequently occurs in patients with cancer. In 2011, Fearon et al proposed that “cancer cachexia is defined as a multifactorial syndrome defined by an ongoing loss of skeletal muscle mass (with or without loss of fat mass) that cannot be fully reversed by conventional nutritional support and leads to progressive functional impairment.”^1^ Cancer cachexia is associated with markedly increased toxicity from chemotherapy,^2^ and significantly impairs the patient's quality of life (QOL).^3^

The mechanism underlying cancer cachexia is not well understood, but various factors are thought to be intricately involved in its development. Inflammatory cytokines produced by cancer cells, such as interleukin (IL)‐1, IL‐6, and tumor necrosis factor‐α, induce skeletal muscle loss, lipolysis, and anorexia, suggesting cancer‐associated inflammation is the main pathogenesis of cancer cachexia.^4^, ^5^ Other metabolic modulators include the dysregulated pituitary–adrenal axis, activated sympathetic nervous system, and adipose tissue browning.^6^ These changes in metabolism are responsible for increased energy expenditure and excessive tissue wasting. Although some guidelines for the management of cancer cachexia were recently published,^7^, ^8^ the pharmacologic treatment options for cancer cachexia are still limited.

Ghrelin is a peptide hormone produced by endocrine cells in the stomach, and acts as a regulator of hunger and a growth hormone (GH) secretagogue.^9^, ^10^, ^11^ In humans, administration of acylated ghrelin stimulated GH release and tended to increase hunger sensations.^12^ Therefore, ghrelin or ghrelin mimetics have been evaluated as possible treatments of cancer cachexia.^7^, ^8^, ^12^

Anamorelin (ONO‐7643) is an orally active, highly selective agonist for the ghrelin receptor.^13^, ^14^, ^15^ Previous Phase 1 and 2 studies^16^, ^17^, ^18^ evaluated the safety and efficacy of anamorelin and showed that it increased body weight, lean body mass (LBM), and food intake. Global phase 3 studies in patients with non‐small cell lung cancer (NSCLC; ROMANA‐1 and ROMANA‐2)^19^ and Japanese studies in patients with NSCLC (ONO‐7643‐03 and ONO‐7643‐04)^20^, ^21^ or gastrointestinal cancers (colorectal cancer, gastric cancer, pancreatic cancer; ONO‐7643‐05)^22^ have also demonstrated beneficial effects of anamorelin on LBM and anorexia. These studies led to the approval of anamorelin in Japan in January 2021 for the management of cachexia in patients with NSCLC, gastric cancer, pancreatic cancer, and colorectal cancer.^23^

To date, the efficacy and safety of anamorelin in patients subgrouped by baseline characteristics have not been well established. Although subgroup analyses of the ROMANA‐1 and ROMANA‐2 studies^19^ showed that anamorelin increased LBM regardless of patient characteristics, those analyses were limited to the changes in LBM. To gain more insight, we performed post‐hoc subgroup analyses of the ONO‐7643‐04 study^20^ of Japanese patients with NSCLC and cachexia to evaluate the effects of anamorelin on body weight, appetite, and LBM across a wide range of subgroups, as well as safety in patients subgrouped by age and performance status. We also compared the LBM, body weight, and appetite responder rates between anamorelin and placebo in various patient subgroups.

## 2 METHODS

The design of the ONO‐7643 study (JapicCTI‐142451) is described in more detail in the previous report.^20^ In brief, the ONO‐7643‐04 study was a randomized, double‐blind, placebo‐controlled, multicenter study for patients with unresectable stage III/IV NSCLC and cachexia in Japan. The primary endpoint was the change from the baseline LBM (measured by dual‐energy x‐ray absorptiometry [DEXA]) over 12 weeks.

### 2.1 Ethics

This study complied with the Declaration of Helsinki and Good Clinical Practice, and it was approved by the ethics committees at the 43 participating centers. All patients provided written informed consent.

### 2.2 Patients and study design

Patients with stage III or IV NSCLC aged ≥20 years were randomized to receive 100 mg anamorelin or placebo once daily in the fasting state (1 h before breakfast) in a double‐blind manner for 12 weeks. Anamorelin was prescribed as a treatment to prevent the progression of cancer cachexia. The eligibility criteria and patient characteristics are summarized in the Supporting Information (Data S1; Table S1) and the prior report.^20^

We used the following endpoints: LBM (as determined by DEXA), body weight, and the QOL questionnaire for cancer patients treated with anticancer drugs (QOL‐ACD), assessed at baseline and at weeks 1 (except DEXA), 3, 6, 9, and 12. Either the General Electric Lunar system (General Electric, Wauwatosa, Wisconsin) or the Hologic system (Hologic, Bedford, Massachusetts) were used to measure LBM.

QOL‐ACD comprised four domains (functional, physical, mental, and psychosocial) across 22 items, each of which is scored on a scale of 1–5, where 5 = best response and 1 = worst response.^24^ Item 8, “Did you have a good appetite,” was used in the present analyses to evaluate the patient's perceived appetite.^24^, ^25^

Safety was recorded throughout the study in terms of adverse events (AEs), adverse drug reactions (ADRs), laboratory tests, vital signs, and 12‐lead electrocardiography.

### 2.3 Patient subgroups

Patients were divided into the following subgroups to assess the efficacy of anamorelin versus placebo: sex (male, female); age (<65, ≥65 years); body mass index (BMI) (<20, ≥20 kg/m^2^); body weight loss within the last 6 months (≥5% to <10%, ≥10%); Eastern Cooperative Oncology Group Performance Status (ECOG PS) (0–1, 2); concomitant anticancer therapy (none, chemotherapy excluding epidermal growth factor receptor tyrosine kinase inhibitors [EGFR‐TKI]); number of prior chemotherapy regimens (1, 2, 3, or ≥4); C‐reactive protein (≤5.0, >5.0 mg/L); albumin (<3.2, ≥3.2 g/dL); and hemoglobin (<12, ≥12 g/dL). Patients were also divided into the following subgroups for the safety analysis: age (<65, ≥65 to <75, ≥75 years) and ECOG PS (0–1, 2).

### 2.4 Outcomes

For these subgroup analyses of efficacy, the outcomes were the mean changes in LBM, body weight, and QOL‐ACD item 8 scores over 12 weeks from baseline, the responder rates, and the odds ratios (ORs) among the specified subgroups. LBM responders were defined as patients whose LBM was maintained or increased (≥0 kg) from baseline to all evaluation time points. Body weight responders were defined as patients whose body weight was maintained or increased (≥0 kg) from baseline to all evaluation time points. Appetite responders were defined as patients whose QOL‐ACD item 8 score was maintained or increased (≥0 point) from baseline to all evaluation time points. Safety was evaluated in terms of AEs and ADRs in groups of patients by age (<65, ≥65 to <75, ≥75 years) and ECOG PS (0–1, 2).

### 2.5 Statistical analyses

Patient characteristics are reported descriptively, as the mean ± standard deviation or number (percent) of patients. The least‐squares (LS) mean changes with 95% confidence intervals (CI) in LBM, body weight, and appetite scores from baseline were analyzed using mixed‐effects models. Responder rates were compared between subgroups using ORs with 95% CIs. The statistical analyses are described in more detail in Data S1. No adjustment was made for multiple comparisons. *p* < 0.05 was considered statistically significant.

## 3 RESULTS

### 3.1 Patients

As previously described, 90 patients received placebo and 83 received anamorelin during the 12‐week treatment period, of which 63 and 55, respectively, completed the study. The baseline characteristics of both groups of patients were generally well matched (Table S1).

### 3.2 Changes in LBM by baseline characteristics of patients

**FIGURE 1:**
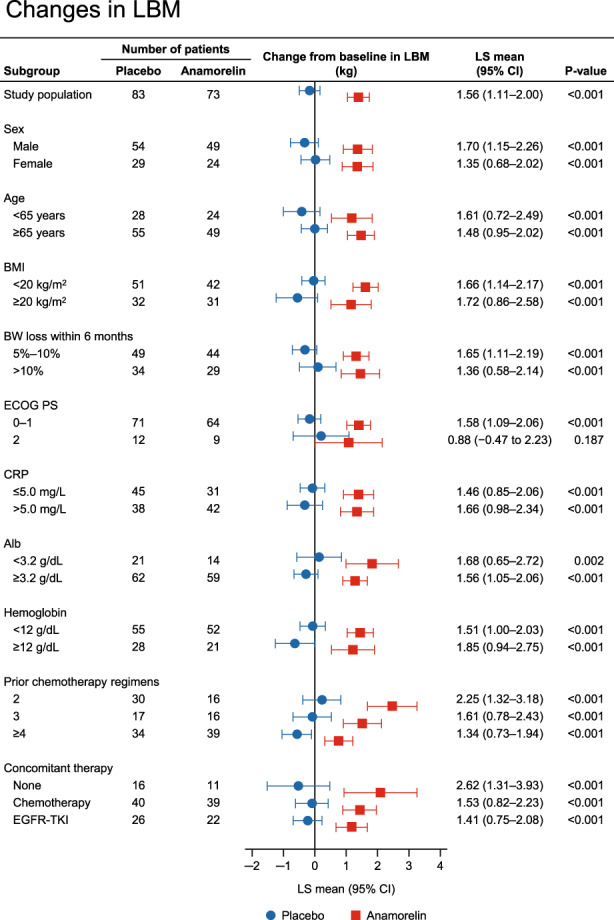
Subgroup analysis of changes in LBM at 12 weeks according to sex, age, Alb, albumin; BMI, BW loss within 6 months, ECOG PS, and concomitant anticancer therapy. BMI, body mass index; BW, body weight; CI, confidence interval; CRP, C‐reactive protein; ECOG PS, Eastern Cooperative Oncology Group Performance Status; EGFR‐TKI, epidermal growth factor receptor tyrosine kinase inhibitor; LBM, lean body weight; LS, least‐squares; OR, odds ratio.

The changes in LBM by various baseline characteristics are shown in Figure 1. The changes in LBM from baseline to week 12 were significantly greater with anamorelin than with placebo in almost all subgroups of patients. The change in LBM was not significantly different in the PS 2 subgroup (*p* = 0.187).

### 3.3 Changes in body weight by baseline characteristics of patients

**FIGURE 2:**
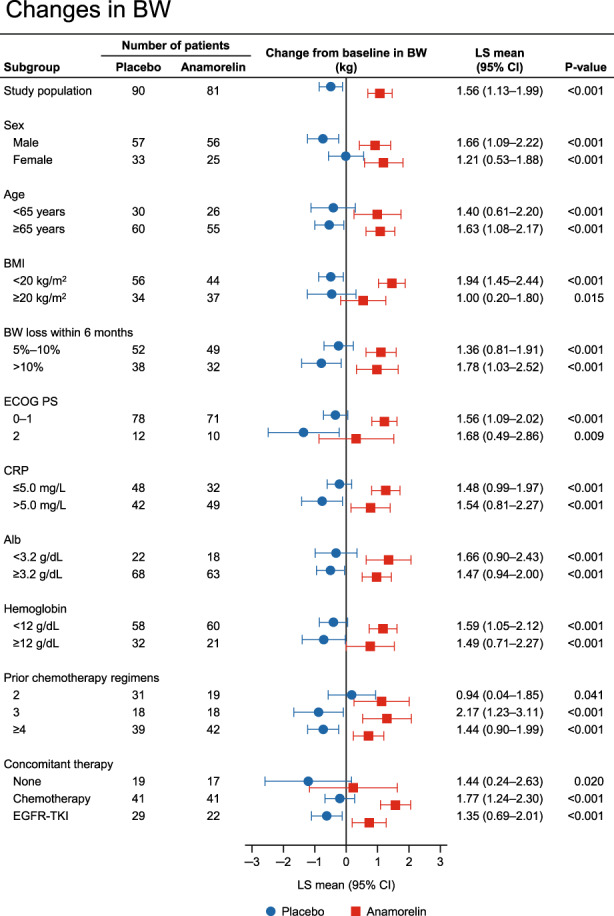
Subgroup analysis of changes in BW at 12 weeks according to sex, age, Alb, albumin; BMI, BW loss within 6 months, ECOG PS, and concomitant anticancer therapy. BMI, body mass index; BW, body weight; CI, confidence interval; CRP, C‐reactive protein; ECOG PS, Eastern Cooperative Oncology Group Performance Status; EGFR‐TKI, epidermal growth factor receptor tyrosine kinase inhibitor; LS, least‐squares; OR, odds ratio.

The changes in body weight from baseline to week 12 are compared between anamorelin and placebo in each subgroup in Figure 2. Similar to the results for LBM, the changes in body weight were consistently greater with anamorelin than with placebo in all subgroups of patients.

### 3.4 Changes in appetite (QOL‐ACD item 8 score) by baseline characteristics of patients

**FIGURE 3:**
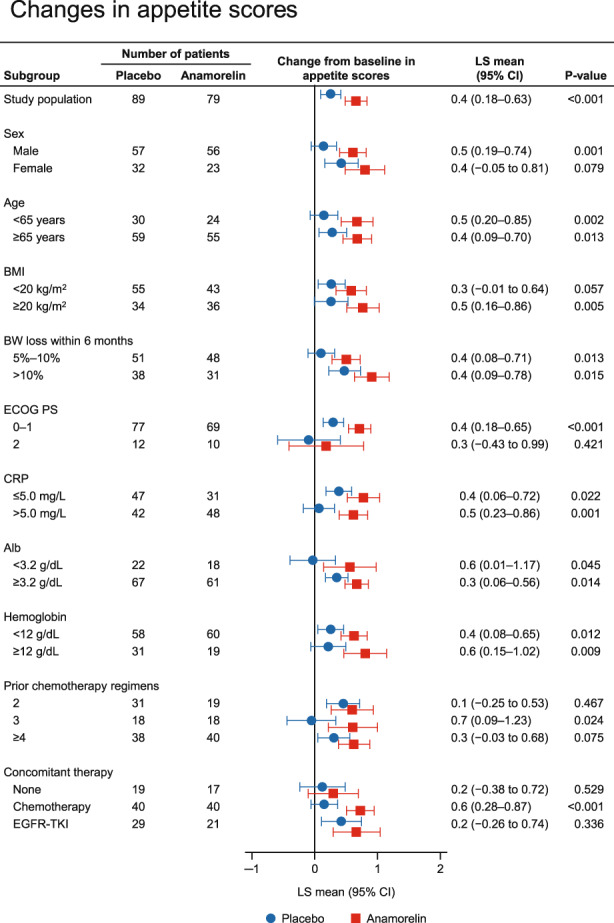
Subgroup analysis of changes in appetite (QOL‐ACD item 8) scores at 12 weeks according to sex, age, Alb, albumin; BMI, BW loss within 6 months, ECOG PS, and concomitant anticancer therapy. BMI, body mass index; BW, body weight; CI, confidence interval; CRP, C‐reactive protein; ECOG PS, Eastern Cooperative Oncology Group Performance Status; EGFR‐TKI, epidermal growth factor receptor tyrosine kinase inhibitor; LS, least‐squares; OR, odds ratio; QOL‐ACD, quality of life questionnaire for cancer patients treated with anticancer drugs.

We also compared the changes in appetite scores from baseline to week 12 between anamorelin and placebo in each subgroup, and the results are shown in Figure 3. The changes in QOL‐ACD item 8 score were consistently greater with anamorelin than with placebo in all subgroups of patients.

### 3.5 Comparison of responder rates between anamorelin and placebo by baseline characteristics of patients

**FIGURE 4:**
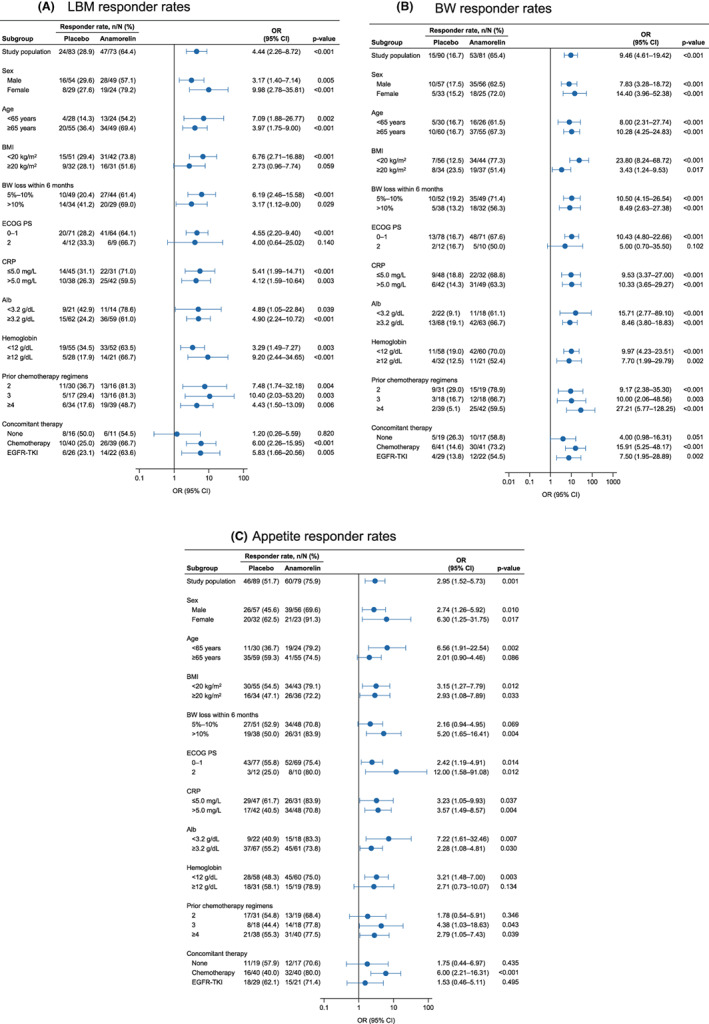
Subgroup analysis of LBM (A), body weight (B), and appetite (QOL‐ACD item 8; C) responder rates according to sex, age, Alb, albumin; BMI, BW loss within 6 months, CRP, C‐reactive protein; ECOG PS, and concomitant anticancer therapy. BMI, body mass index; BW, body weight; CI, confidence interval; ECOG PS, Eastern Cooperative Oncology Group Performance Status; EGFR‐TKI, epidermal growth factor receptor tyrosine kinase inhibitor; LBM, lean body weight; OR, odds ratio; QOL‐ACD, quality of life questionnaire for cancer patients treated with anticancer drugs.

The results of the responder analysis of LBM, body weight, and appetite are shown in Figure 4A–C, respectively. Anamorelin was associated with greater responder rates than placebo for LBM, body weight, and appetite in most of the subgroups.

### 3.6 Safety by age and ECOG PS

The overall safety outcomes were described in detail in our earlier report.^20^ In this report, we show the tolerability of anamorelin in subgroups of patients by age (<65, ≥65–<75, ≥75 years; Table 1) and ECOG PS (0–1, 2; Table S2). The incidence of ADRs among anamorelin‐treated patients increased with age, with frequencies of 19.2%, 45.9%, and 60.0% in patients aged <65, ≥65 to <75, and ≥75 years, respectively (Table 1). Of the 20 anamorelin‐treated patients aged ≥75 years, four had grade 1 ADRs, six had grade 2 ADRs, and two had grade 3 ADRs. ADRs that appeared to be more frequent (>2 patients) in older patients (≥75 years) included rash, first‐degree atrioventricular block, diabetes mellitus, headache, and hot flush (Table S3). Table S4 shows the frequencies of ADRs in patients divided by ECOG PS. In the anamorelin group, ADRs tended to be more frequent in the PS 2 subgroup (60.0%) than the PS 0–1 subgroup (38.4%). The frequency of serious ADRs was 2.7% in the PS 0–1 subgroup and 0% in the PS 2 subgroup. There were no specific ADRs with a higher frequency in the PS 2 subgroup than in the PS 0–1 subgroup. There were no deaths due to AEs during the study period in any age or ECOG PS subgroup.

**TABLE 1:** Overview of adverse events and adverse drug reactions in age subgroups (safety analysis set)

	No. of patients (%)^a^					
	<65 years		≥65 to <75 years		≥75 years	
	Placebo	Anamorelin	Placebo	Anamorelin	Placebo	Anamorelin
	(*N* = 30)	(*N* = 26)	(*N* = 48)	(*N* = 37)	(*N* = 12)	(*N* = 20)
AEs	26 (86.7)	23 (88.5)	37 (77.1)	34 (91.9)	10 (83.3)	17 (85.0)
Difference vs. placebo, % (95% CI)	1.8 (−15.5 to 19.1)		14.8 (0.0 to 29.6)		1.7 (−24.6 to –27.9)	
*p*‐value	0.840		0.068		0.900	
*p*‐value among anamorelin subgroups^b^	0.720					
Serious AEs	4 (13.3)	6 (23.1)	4 (8.3)	7 (18.9)	0	3 (15.0)
Discontinuations due to AEs	0	0	2 (4.2)	1 (2.7)	0	2 (10.0)
ADRs	7 (23.3)	5 (19.2)	12 (25.0)	17 (45.9)	1 (8.3)	12 (60.0)
Difference vs. placebo, % (95% CI)	−4.1 (−25.5 to 17.3)		20.9 (0.7 to 41.1)		51.7 (25.1 to 78.2)	
*p*‐value	0.709		0.043		0.004	
*p*‐value among anamorelin subgroups^b^	0.015					
Serious ADRs	0	1 (3.8)	0	1 (2.7)	0	0
Discontinuations due to ADRs	0	0	1 (2.1)	1 (2.7)	0	1 (5.0)
Deaths	0	0	0	0	0	0
ADRs by grade						
1	4 (13.3)	2 (7.7)	10 (20.8)	4 (10.8)	0	4 (20.0)
2	1 (3.3)	1 (3.8)	2 (4.2)	11 (29.7)	1 (8.3)	6 (30.0)
3	2 (6.7)	2 (7.7)	0	2 (5.4)	0	2 (10.0)

Abbreviations: AEs, adverse events; CI, confidence interval; ADRs, adverse drug reactions.

^a^ Unless indicated.

^b^ *p*‐values for the comparisons of frequencies of AEs/ADRs among the subgroups by age and PS.

## 4 DISCUSSION

We performed analyses of the ONO‐7643‐04 study to evaluate the efficacy and safety of anamorelin in subgroups. In terms of its efficacy, we found greater improvements in LBM, body weight, and appetite with anamorelin than with placebo for nearly all the analyzed subgroups. Overall, these results suggest that the efficacy of anamorelin is unaffected by most patient characteristics. Furthermore, anamorelin may have benefits in terms of LBM gain, weight gain, and improved appetite regardless of the patient's nutrition‐related biomarkers at baseline, including C‐reactive protein, albumin, and hemoglobin.

The results regarding the LBM change in the subgroups in our study are generally similar to those in the international ROMANA‐1 and ROMANA‐2 studies.^19^ Anamorelin had a marked effect on LBM compared with placebo regardless of the patient's sex, age, BMI, prior weight loss, PS, and concomitant anticancer therapy (Figure 1). In addition, an effect of anamorelin was observed in the PS 2 subgroup, albeit with numerically smaller LS means compared with the PS 0–1 subgroup, although the sample size in both clinical trials was limited. Furthermore, in subgroups of patients according to number of prior chemotherapy regimens, which was not analyzed in the ROMANA‐1 and ROMANA‐2 studies, we found that the LS means for anamorelin were greater in patients with a history of fewer chemotherapy regimens, suggesting that anamorelin may be more effective when used in earlier treatment lines. Similar to the results of the LBM analysis, the changes in body weight from baseline to week 12 were significantly greater with anamorelin than with placebo in all subgroups of patients (Figure 2). It is well known that weight loss has deleterious effects on the efficacy of anticancer therapies.^26^, ^27^, ^28^ Therefore, our results suggest that anamorelin may be useful for a wide range of patients showing weight loss, although the influence of anamorelin on cancer therapy should be examined in more detail in future studies.

The changes in appetite scores from baseline to week 12 were greater with anamorelin than with placebo in most subgroups of patients, although the difference between the anamorelin and placebo groups was attenuated in the following subgroups: female, low BMI, PS 2, no concomitant chemotherapy, concomitant chemotherapy with EGFR‐TKI, and history of two prior chemotherapy regimens (Figure 3). Appetite is a subjective symptom and is difficult to evaluate. In this study, we used item 8 of the QOL‐ACD, “Did you have a good appetite?”, to assess appetite. Because a minimal important difference has not yet been established using the QOL‐ACD due to limited validation, further clinical trials using QOL‐ACD or other well‐validated assessment methods are needed to confirm our finding.

The ORs for LBM and body weight responder rates tended to be greater in patients with low BMI at baseline than in patients with higher BMI (Figure 4A,B). The results of a pharmacokinetic study indicated that the apparent total clearance of anamorelin was slower in patients with low body weight,^29^ which may result in greater exposure in those patients. Because a similar effect may occur in patients with low BMI, greater exposure may contribute to greater increases in body weight and LBM.

Regarding the appetite responder rate, the OR tended to be greater in patients with PS 2 than in those with ECOG PS 0–1 (Figure 4C). Previous studies have revealed that weight loss is associated with anorexia symptoms and PS^30^ and patients with cancer cachexia tended to have a worse general condition (as measured by the Karnofsky performance status).^31^ Patients with PS 2 may have more severe anorexia at baseline than patients with PS 0–1. Patients with severe anorexia may recognize a greater improvement of appetite than other patients. The OR for the appetite responder rate was relatively high in patients with concomitant chemotherapy compared with patients without concomitant anticancer treatment or those treated with concomitant EGFR‐TKIs. Chemotherapy is associated with high rates of nausea and vomiting, leading to onset and aggravation of anorexia. Chemotherapy‐induced nausea and vomiting is also associated with cancer cachexia.^26^, ^32^ Patients with nausea and vomiting associated with chemotherapy may recognize a greater improvement in their appetite. Weight loss and decreased appetite are among the most frequent side effects of anticancer treatment. Much effort has been made to help decrease these side effects, with many therapies under evaluation (e.g.,^33^, ^34^, ^35^, ^36^, ^37^). Our findings demonstrate that anamorelin is a useful treatment option for patients with cancer cachexia.

Adverse drug reactions are of particular concern in elderly patients or those with poor general condition. Notably, the incidences of cardiac conduction disorders and blood glucose‐related disorders, which were more frequent in anamorelin‐treated patients,^20^ may be affected by age and PS. Although the incidence of ADRs increased with age and PS, the incidence of serious ADRs did not increase in these subgroups. In addition, ADRs observed in elderly patients (≥75 years) or patients with PS 2 were mostly grade 1 or 2. These results suggest that the tolerability of anamorelin is not influenced by age or PS.

Limitations of this study include its design and setting as a controlled clinical trial with strict eligibility criteria. Thus, the results may not fully represent its use in actual clinical practice. The study excluded patients with PS ≥3. Therefore, we could not evaluate the efficacy or safety of anamorelin in these patients. Some subgroups had small numbers of patients with high variability, and no adjustment was made for multiple comparisons. These factors may limit the robustness of the statistical analyses and raise the possibility of type II error. In addition, we used different methods to measure body weight (scales) and LBM (DEXA), which may contribute to the differing gains in body weight and LBM. Furthermore, the QOL‐ACD has not been sufficiently validated for the assessment of appetite, and no minimal important difference has been set. In international studies, the Functional Assessment of Anorexia/Cachexia Treatment, which consists of multiple items, was used to assess appetite. In the present study, we evaluated appetite using a single question (item 8 of QOL‐ACD). This item was used in the previous clinical trials of anamorelin in Japan with consistent results among the trials.^20^, ^21^, ^22^, ^38^ Therefore, we believe this item provides a reliable assessment of appetite.

In conclusion, we observed greater improvements in LBM, body weight, and appetite, together with higher responder rates, in anamorelin‐treated patients compared with placebo‐treated patients in nearly all subgroups analyzed in this study of patients with NSCLC and cancer cachexia. We also found that anamorelin is tolerable regardless of age or PS. Taken together, these results suggest that anamorelin has a beneficial impact on cancer treatment by maintaining and improving the condition of cancer patients through improvements in body weight and appetite that are regardless of patient characteristics.

## AUTHOR CONTRIBUTIONS

Koichi Takayama: Conceptualization, study design, data interpretation, and writing—drafting, critical review, and final approval of the manuscript. Toru Takiguchi: Conceptualization, study design, data interpretation, writing—drafting, critical review, and final approval of the manuscript. Naoyuki Komura: Conceptualization, study design, data interpretation, writing—drafting, critical review, and final approval of the manuscript. Tateaki Naito: Conceptualization, study design, data collection, data interpretation, writing—drafting, critical review, and final approval of the manuscript.

## FUNDING INFORMATION

This study was funded by Ono Pharmaceutical Co., Ltd.

## CONFLICT OF INTEREST

Koichi Takayama reports research grants to the institution from Ono Pharmaceutical, Taiho Pharmaceutical, Eli‐Lilly, and Fukuda Lifetech; consulting fees from Ono Pharmaceutical; lecture fees from Ono Pharmaceutical, AstraZeneca, MSD, Chugai‐Roche, Eli‐Lilly, and Boehringer‐Ingelheim; and travel expenses from Ono Pharmaceutical beyond the work described in this manuscript. Toru Takiguchi and Naoyuki Komura are employees of Ono Pharmaceutical Co., Ltd. Tateaki Naito reports research funding to the institution from Ono Pharmaceutical and Otsuka Pharmaceutical; and lecture fees and travel expenses from Ono Pharmaceutical beyond the work described in this manuscript.

## ETHICS APPROVAL

This study complied with the Declaration of Helsinki and Good Clinical Practice, and it was approved by the ethics committees at the 43 participating centers. All patients provided written informed consent.

## STUDY REGISTRATION

JapicCTI‐142451 (Japan Pharmaceutical Information Center).

## PRIOR PUBLICATION

Katakami N, Uchino J, Yokoyama T, et al. Anamorelin (ONO‐7643) for the treatment of patients with non–small cell lung cancer and cachexia: results from a randomized, double‐blind, placebo‐controlled, multicenter study of Japanese patients (ONO‐7643‐04). *Cancer*. 2018;124(3):606–616. Results of the subgroup analyses and responder rates were submitted as abstracts to the 62nd Annual Meeting of the Japan Lung Cancer Society, November 26–28, 2021, and the 2022 Japanese Society of Medical Oncology Annual Meeting, February 17–19, 2022.

## Supporting information


Data S1; Tables S1–S4

## Data Availability

Qualified researchers may request Ono Pharmaceutical Co., Ltd. to disclose individual patient‐level data from clinical studies through the following website: https://www.clinicalstudydatarequest.com/. For more information on Ono Pharmaceutical Co., Ltd.’s Policy for the Disclosure of Clinical Study Data, please see the following website: https://www.ono.co.jp/eng/rd/policy.html.

## References

[1] Fearon K , Strasser F , Anker SD , et al. Definition and classification of cancer cachexia: an international consensus. Lancet Oncol. 2011;12(5):489‐495. 21296615 10.1016/S1470-2045(10)70218-7

[2] Fearon K , Arends J , Baracos V . Understanding the mechanisms and treatment options in cancer cachexia. Nat Rev Clin Oncol. 2013;10(2):90‐99. 23207794 10.1038/nrclinonc.2012.209

[3] LeBlanc TW , Nipp RD , Rushing CN , et al. Correlation between the international consensus definition of the cancer anorexia‐cachexia syndrome (CACS) and patient‐centered outcomes in advanced non‐small cell lung cancer. J Pain Symptom Manage. 2015;49(4):680‐689. 25461669 10.1016/j.jpainsymman.2014.09.008

[4] Aoyagi T , Terracina KP , Raza A , Matsubara H , Takabe K . Cancer cachexia, mechanism and treatment. World J Gastrointest Oncol. 2015;7(4):17‐29. 25897346 10.4251/wjgo.v7.i4.17 PMC4398892

[5] Evans WJ , Morley JE , Argilés J , et al. Cachexia: a new definition. Clin Nutr. 2008;27(6):793‐799. 18718696 10.1016/j.clnu.2008.06.013

[6] Baazim H , Antonio‐Herrera L , Bergthaler A . The interplay of immunology and cachexia in infection and cancer. Nat Rev Immunol. 2022;22(5):309‐321. 34608281 10.1038/s41577-021-00624-w PMC8489366

[7] Arends J , Strasser F , Gonella S , et al. Cancer cachexia in adult patients: ESMO Clinical Practice Guidelines. ESMO Open. 2021;6(3):100092. 34144781 10.1016/j.esmoop.2021.100092 PMC8233663

[8] Roeland EJ , Bohlke K , Baracos VE , et al. Management of cancer cachexia: ASCO guideline. J Clin Oncol. 2020;38(21):2438‐2453. 32432946 10.1200/JCO.20.00611

[9] Kojima M , Hosoda H , Date Y , Nakazato M , Matsuo H , Kangawa K . Ghrelin is a growth‐hormone‐releasing acylated peptide from stomach. Nature. 1999;402(6762):656‐660. 10604470 10.1038/45230

[10] Neary NM , Small CJ , Wren AM , et al. Ghrelin increases energy intake in cancer patients with impaired appetite: acute, randomized, placebo‐controlled trial. J Clin Endocrinol Metab. 2004;89(6):2832‐2836. 15181065 10.1210/jc.2003-031768

[11] Wren AM , Seal LJ , Cohen MA , et al. Ghrelin enhances appetite and increases food intake in humans. J Clin Endocrinol Metab. 2001;86(12):5992. 11739476 10.1210/jcem.86.12.8111

[12] Akamizu T , Takaya K , Irako T , et al. Pharmacokinetics, safety, and endocrine and appetite effects of ghrelin administration in young healthy subjects. Eur J Endocrinol. 2004;150(4):447‐455. 15080773 10.1530/eje.0.1500447

[13] Zhang H , Garcia JM . Anamorelin hydrochloride for the treatment of cancer‐anorexia‐cachexia in NSCLC. Expert Opin Pharmacother. 2015;16(8):1245‐1253. 25945893 10.1517/14656566.2015.1041500 PMC4677053

[14] Currow DC , Abernethy AP . Anamorelin hydrochloride in the treatment of cancer anorexia‐cachexia syndrome. Future Oncol. 2014;10(5):789‐802. 24472001 10.2217/fon.14.14

[15] Pietra C , Takeda Y , Tazawa‐Ogata N , et al. Anamorelin HCl (ONO‐7643), a novel ghrelin receptor agonist, for the treatment of cancer anorexia‐cachexia syndrome: preclinical profile. J Cachexia Sarcopenia Muscle. 2014;5(4):329‐337. 25267366 10.1007/s13539-014-0159-5 PMC4248409

[16] Garcia JM , Friend J , Allen S . Therapeutic potential of anamorelin, a novel, oral ghrelin mimetic, in patients with cancer‐related cachexia: a multicenter, randomized, double‐blind, crossover, pilot study. Support Care Cancer. 2013;21(1):129‐137. 22699302 10.1007/s00520-012-1500-1

[17] Garcia JM , Polvino WJ . Effect on body weight and safety of RC‐1291, a novel, orally available ghrelin mimetic and growth hormone secretagogue: results of a phase I, randomized, placebo‐controlled, multiple‐dose study in healthy volunteers. Oncologist. 2007;12(5):594‐600. 17522248 10.1634/theoncologist.12-5-594

[18] Garcia JM , Polvino WJ . Pharmacodynamic hormonal effects of anamorelin, a novel oral ghrelin mimetic and growth hormone secretagogue in healthy volunteers. Growth Horm IGF Res. 2009;19(3):267‐273. 19196529 10.1016/j.ghir.2008.12.003

[19] Temel JS , Abernethy AP , Currow DC , et al. Anamorelin in patients with non‐small‐cell lung cancer and cachexia (ROMANA 1 and ROMANA 2): results from two randomised, double‐blind, phase 3 trials. Lancet Oncol. 2016;17(4):519‐531. 26906526 10.1016/S1470-2045(15)00558-6

[20] Katakami N , Uchino J , Yokoyama T , et al. Anamorelin (ONO‐7643) for the treatment of patients with non‐small cell lung cancer and cachexia: results from a randomized, double‐blind, placebo‐controlled, multicenter study of Japanese patients (ONO‐7643‐04). Cancer. 2018;124(3):606‐616. 29205286 10.1002/cncr.31128 PMC5814824

[21] Takayama K , Katakami N , Yokoyama T , et al. Anamorelin (ONO‐7643) in Japanese patients with non‐small cell lung cancer and cachexia: results of a randomized phase 2 trial. Support Care Cancer. 2016;24(8):3495‐3505. 27005463 10.1007/s00520-016-3144-z PMC4917578

[22] Hamauchi S , Furuse J , Takano T , et al. A multicenter, open‐label, single‐arm study of anamorelin (ONO‐7643) in advanced gastrointestinal cancer patients with cancer cachexia. Cancer. 2019;125(23):4294‐4302. 31415709 10.1002/cncr.32406 PMC6900019

[23] Wakabayashi H , Arai H , Inui A . The regulatory approval of anamorelin for treatment of cachexia in patients with non‐small cell lung cancer, gastric cancer, pancreatic cancer, and colorectal cancer in Japan: facts and numbers. J Cachexia Sarcopenia Muscle. 2021;12(1):14‐16. 33382205 10.1002/jcsm.12675 PMC7890143

[24] Kurihara M , Shimizu H , Tsuboi K , et al. Development of quality of life questionnaire in Japan: quality of life assessment of cancer patients receiving chemotherapy. Psychooncology. 1999;8(4):355‐363. 10474853 10.1002/(SICI)1099-1611(199907/08)8:4<355::AID-PON401>3.0.CO;2-I

[25] Matsumoto T , Ohashi Y , Morita S , et al. The quality of life questionnaire for cancer patients treated with anticancer drugs (QOL‐ACD): validity and reliability in Japanese patients with advanced non‐small‐cell lung cancer. Qual Life Res. 2002;11(5):483‐493. 12113395 10.1023/a:1015614505929

[26] da Rocha IMG , Marcadenti A , de Medeiros GOC , et al. Is cachexia associated with chemotherapy toxicities in gastrointestinal cancer patients? A prospective study. J Cachexia Sarcopenia Muscle. 2019;10(2):445‐454. 30924270 10.1002/jcsm.12391 PMC6463470

[27] Fujii H , Makiyama A , Iihara H , et al. Cancer cachexia reduces the efficacy of nivolumab treatment in patients with advanced gastric cancer. Anticancer Res. 2020;40(12):7067‐7075. 33288604 10.21873/anticanres.14734

[28] Morimoto K , Uchino J , Yokoi T , et al. Impact of cancer cachexia on the therapeutic outcome of combined chemoimmunotherapy in patients with non‐small cell lung cancer: a retrospective study. Oncoimmunology. 2021;10(1):1950411. 34290909 10.1080/2162402X.2021.1950411 PMC8274442

[29] Adlumiz® (Anamorelin) , Common Technical Document, 2011. Available at: https://www.pmda.go.jp/drugs/2021/P20210113006/180188000_30300AMX00003_K100_1.pdf [In Japanese]. Last accessed October 4, 2021.

[30] Mariani L , Lo Vullo S , Bozzetti F . Weight loss in cancer patients: a plea for a better awareness of the issue. Support Care Cancer. 2012;20(2):301‐309. 21210155 10.1007/s00520-010-1075-7

[31] LeBlanc TW , Samsa GP , Wolf SP , Locke SC , Cella DF , Abernethy AP . Validation and real‐world assessment of the functional assessment of anorexia‐cachexia therapy (FAACT) scale in patients with advanced non‐small cell lung cancer and the cancer anorexia‐cachexia syndrome (CACS). Support Care Cancer. 2015;23(8):2341‐2347. 25586527 10.1007/s00520-015-2606-z

[32] Navari RM . Nausea and vomiting in advanced cancer. Curr Treat Options Oncol. 2020;21(2):14. 32025954 10.1007/s11864-020-0704-8

[33] Hashimoto H , Abe M , Tokuyama O , et al. Olanzapine 5 mg plus standard antiemetic therapy for the prevention of chemotherapy‐induced nausea and vomiting (J‐FORCE): a multicentre, randomised, double‐blind, placebo‐controlled, phase 3 trial. Lancet Oncol. 2020;21(2):242‐249. 31838011 10.1016/S1470-2045(19)30678-3

[34] Hesketh PJ , Grunberg SM , Gralla RJ , et al. The oral neurokinin‐1 antagonist aprepitant for the prevention of chemotherapy‐induced nausea and vomiting: a multinational, randomized, double‐blind, placebo‐controlled trial in patients receiving high‐dose cisplatin‐‐the Aprepitant Protocol 052 Study Group. J Clin Oncol. 2003;21(22):4112‐4119. 14559886 10.1200/JCO.2003.01.095

[35] Moezian GSA , Javadinia SA , Sales SS , Fanipakdel A , Elyasi S , Karimi G . Oral silymarin formulation efficacy in management of AC‐T protocol induced hepatotoxicity in breast cancer patients: a randomized, triple blind, placebo‐controlled clinical trial. J Oncol Pharm Pract. 2021;28(4):827‐835. 33861657 10.1177/10781552211006182

[36] Salek R , Dehghani M , Mohajeri SA , Talaei A , Fanipakdel A , Javadinia SA . Amelioration of anxiety, depression, and chemotherapy related toxicity after crocin administration during chemotherapy of breast cancer: a double blind, randomized clinical trial. Phytother Res. 2021;35(9):5143‐5153. 34164855 10.1002/ptr.7180

[37] Sedighi Pashaki A , Mohammadian K , Afshar S , et al. A randomized, controlled, parallel‐group, trial on the effects of melatonin on fatigue associated with breast cancer and its adjuvant treatments. Integr Cancer Ther. 2021;20:1534735420988343. 33543655 10.1177/1534735420988343 PMC7868453

[38] Naito T , Uchino J , Kojima T , et al. A multicenter, open‐label, single‐arm study of anamorelin (ONO‐7643) in patients with cancer cachexia and low body mass index. Cancer. 2022;128:2025‐2035. 35195274 10.1002/cncr.34154 PMC9303784

