# Supplementary material for: Efficacy and safety of anamorelin in patients with cancer cachexia: Post‐hoc subgroup analyses of a placebo‐controlled study
Source: Cancer Med. 2022 Nov 16;12(3):2918–28. doi: 10.1002/cam4.5206 (PMC9939183; doi:10.1002/cam4.5206)
Supplement: Supplementary file 1 — Data S1; Tables S1–S4 [file CAM4-12-2918-s001.pdf]

SUPPORTING INFORMATION

Efficacy and safety of anamorelin in patients with cancer cachexia: Post-hoc subgroup analyses of a placebo-controlled study

Koichi Takayama, Toru Takiguchi, Naoyuki Komura, Tateaki Naito

Contents

	Page
**Data S1: Supplemental methods.** Eligibility criteria, statistical analyses	2
**Table S1.** Patient characteristics	4
**Table S2.** Overview of adverse events and adverse drug reactions by ECOG PS (safety analysis set)	6
**Table S3.** Adverse drug reactions in >2% of patients by age (safety analysis set)	7
**Table S4.** Adverse drug reactions in >2% of patients by ECOG PS (safety analysis set)	10

Data S1: Supplemental methods

Eligibility criteria

As previously described,[1] patients were eligible for this study if they had experienced involuntary weight loss of ≥5% within the last 6 months, anorexia, and two or more relevant symptoms (fatigue, malaise, reduced overall muscular strength, arm muscle circumference <10th percentile, in centimeters), plus more than one of the following: albumin <3.2 g/dL, C-reactive protein >5.0 mg/L, hemoglobin <12 g/dL, ECOG PS of 0–2, and estimated life expectancy of ≥4 months. The symptoms anorexia, fatigue, malaise, and reduced overall muscular strength had to be reported as grade ≥1, based on the National Cancer Institute's Common Terminology Criteria for Adverse Events (version 4.0).

Statistical analyses

Patient characteristics are reported descriptively, as the mean ± standard deviation or number (percent) of patients. Least-squares (LS) mean changes from baseline in LBM, body weight, and appetite scores (QOL-ACD item 8) were calculated for each subgroup using the mixed-effects model for repeated measures together with the LS mean difference for anamorelin versus placebo, with 95% confidence intervals (CI).

The LBM, body weight, and appetite response rates were compared between anamorelin and placebo in each subgroup using ORs with 95% CI. The denominator was the number of patients for whom the parameter was evaluated.

We also determined the LS mean (95% CI) changes in QOL-ACD item 8 scores over time in responders and non-responders for LBM and body weight, and the LS mean (95% CI) changes in LBM over time in responders and non-responders for appetite. Differences at each visit were determined using the mixed-effects model for repeated measures.

The frequencies of AEs and ADRs were compared using χ^2 tests between anamorelin and placebo for patients divided by age and ECOG PS.

Because these analyses were conducted in a post-hoc manner for exploratory purposes, no adjustment was made for multiple comparisons and $P < 0.05$ was considered statistically significant. All analyses were conducted using SAS version 9.4 (SAS Institute, Cary, NC, USA).

Reference

1. Katakami N, Uchino J, Yokoyama T, et al. Anamorelin (ONO-7643) for the treatment of patients with non-small cell lung cancer and cachexia: results from a randomized, double-blind, placebo-controlled, multicenter study of Japanese patients (ONO-7643-04). *Cancer*. 2018;124(3):606-616. doi:10.1002/cncr.31128

TABLE S1 Patient characteristics

Variable	No. of patients (%) or mean ± SD	
	Placebo (*N* = 90)	Anamorelin (*N* = 82)
Sex		
Male	57 (63.3)	57 (69.5)
Female	33 (36.7)	25 (30.5)
Age (years)	67.2 ± 7.9	67.9 ± 9.7
Body weight (kg)	49.73 ± 8.32	52.09 ± 9.40
BMI (kg/m^2)	19.27 ± 2.31	19.76 ± 2.59
BW loss within 6 months		
5%–10%	52 (57.8)	49 (59.8)
>10%	38 (42.2)	33 (40.2)
LBM (kg) (DEXA)	37.06 ± 6.34	38.77 ± 7.04
QOL-ACD	70.9 ± 13.0	74.8 ± 13.1
QOL-ACD item 8	2.9 ± 1.1	3.0 ± 1.0
ECOG PS		
0–1	78 (86.7)	72 (87.8)
2	12 (13.3)	10 (12.2)
Prior chemotherapy regimens		
1	2 (2.2)	2 (2.4)
2	31 (34.4)	20 (24.4)
3	18 (20.0)	18 (22.0)
≥4	39 (43.3)	42 (51.2)
Concomitant anticancer therapy		
None	19 (21.1)	18 (22.0)
Chemotherapy (excluding EGFR-TKI)	41 (45.6)	41 (50.0)
EGFR-TKI	29 (32.2)	22 (26.8)

Stage		
IIIa	1 (1.1)	3 (3.7)
IIIb	11 (12.2)	6 (7.3)
IV	60 (66.7)	47 (57.3)
Recurrent NSCLC	18 (20.0)	26 (31.7)
Histological type of NSCLC		
Adenocarcinoma	71 (78.9)	65 (79.3)
Squamous cell	16 (17.8)	14 (17.1)
Other	1 (1.1)	2 (2.4)
Unknown	2 (2.2)	1 (1.2)
Laboratory data at baseline		
CRP (mg/L)	17.2 ± 29.8	21.5 ± 32.3
Alb (g/dL)	3.6 ± 0.5	3.6 ± 0.6
Hemoglobin (g/dL)	10.9 ± 1.8	10.8 ± 1.9

Abbreviations: SD, standard deviation; BMI, body mass index; BW, body weight; LBM, lean body mass; DEXA, dual-energy x-ray absorptiometry; QOL-ACD, quality of life questionnaire for cancer patients treated with anticancer drugs; ECOG PS, Eastern Cooperative Oncology Group Performance Status; EGFR-TKI, epidermal growth factor receptor tyrosine kinase inhibitor; NSCLC, non-small cell lung cancer; CRP, C-reactive protein; Alb, albumin.

Modified from **Table 1** in Katakami N, Uchino J, Yokoyama T, et al. Anamorelin (ONO-7643) for the treatment of patients with non–small cell lung cancer and cachexia: results from a randomized, double-blind, placebo-controlled, multicenter study of Japanese patients (ONO-7643-04). *Cancer* 2018;124(3):606-616. The full analysis set (FAS), the primary efficacy analysis set, was used in the present article.

TABLE S2 Overview of adverse events and adverse drug reactions by ECOG PS (safety analysis set)

	No. of patients (%)*			
	ECOG PS 0–1		ECOG PS 2	
	Placebo (*N* = 78)	Anamorelin (*N* = 73)	Placebo (*N* = 12)	Anamorelin (*N* = 10)
AEs	62 (79.5)	66 (90.4)	11 (91.7)	8 (80.0)
Difference vs placebo, % (95% CI)	10.9 (−0.3 to 22.1)		−11.7 (−41.0 to 17.6)	
P-value	0.062		0.427	
P-value among anamorelin subgroups†	0.321			
Serious AEs	6 (7.7)	15 (20.5)	2 (16.7)	1 (10.0)
Discontinuations due to AEs	2 (2.6)	3 (4.1)	0	0
ADRs	18 (23.1)	28 (38.4)	2 (16.7)	6 (60.0)
Difference vs placebo, % (95% CI)	15.3 (0.7 to 29.8)		43.3 (6.4 to 80.3)	
P-value	0.041		0.035	
P-value among anamorelin subgroups†	0.192			
Serious ADRs	0	2 (2.7)	0	0
Discontinuations due to ADRs	1 (1.3)	2 (2.7)	0	0
ADRs by grade				
1	12 (15.4)	8 (11.0)	2 (16.7)	2 (20.0)
2	4 (5.1)	15 (20.5)	0	3 (30.0)
3	2 (2.6)	5 (6.8)	0	1 (10.0)

*Unless indicated.

†*P*-values for the comparisons of frequencies of AEs/ADRs among the subgroups by age and PS.

Abbreviations: ECOG PS, Eastern Cooperative Oncology Group Performance Status; AEs, adverse events; CI, confidence interval; ADRs, adverse drug reactions.

TABLE S3 Adverse drug reactions in >2% of patients by age (safety analysis set)

	No. of patients (%)					
	<65 years		≥65 to <75 years		≥75 years	
	Placebo (*N* = 30)	Anamorelin (*N* = 26)	Placebo (*N* = 48)	Anamorelin (*N* = 37)	Placebo (*N* = 12)	Anamorelin (*N* = 20)
Nausea	3 (10.0)	0	2 (4.2)	1 (2.7)	0	0
Rash	0	1 (3.8)	0	2 (5.4)	1 (8.3)	2 (10.0)
Atrioventricular block first degree	0	0	0	3 (8.1)	0	2 (10.0)
Gamma-glutamyltransferase increased	1 (3.3)	1 (3.8)	0	2 (5.4)	0	0
Diabetes mellitus	0	0	0	1 (2.7)	0	2 (10.0)
Diarrhea	2 (6.7)	0	0	0	0	1 (5.0)
Dizziness	1 (3.3)	0	1 (2.1)	0	0	1 (5.0)
Glycosylated hemoglobin increased	0	0	1 (2.1)	1 (2.7)	0	1 (5.0)
Headache	0	0	1 (2.1)	0	0	2 (10.0)
Hyperglycemia	1 (3.3)	1 (3.8)	0	0	0	1 (5.0)
Blood creatinine increased	0	0	2 (4.2)	0	0	0
Dysgeusia	0	1 (3.8)	1 (2.1)	0	0	0
Hot flush	0	0	0	0	0	2 (10.0)
Hypertension	0	1 (3.8)	0	1 (2.7)	0	0
Edema	0	0	0	1 (2.7)	0	1 (5.0)
Edema peripheral	0	0	0	1 (2.7)	0	1 (5.0)
Pyrexia	0	0	0	1 (2.7)	0	1 (5.0)
Tachycardia	0	0	0	2 (5.4)	0	0
Vertigo	0	0	2 (4.2)	0	0	0

Vomiting	0	0	1 (2.1)	1 (2.7)	0	0
Abdominal pain upper	0	0	0	1 (2.7)	0	0
Anemia	1 (3.3)	0	0	0	0	0
Blood bilirubin increased	0	0	0	1 (2.7)	0	0
Blood pressure decreased	0	0	0	1 (2.7)	0	0
Blood triglycerides increased	0	0	1 (2.1)	0	0	0
Blood urine present	0	0	0	1 (2.7)	0	0
Bundle branch block right	0	0	1 (2.1)	0	0	0
Chest pain	0	0	0	0	0	1 (5.0)
Delirium	0	0	0	1 (2.7)	0	0
Dental caries	0	0	0	1 (2.7)	0	0
Dyspnea	0	0	0	1 (2.7)	0	0
Electrocardiogram PR prolongation	0	0	0	0	0	1 (5.0)
Electrocardiogram QRS complex prolonged	0	0	0	1 (2.7)	0	0
Electrocardiogram QT prolonged	0	0	1 (2.1)	0	0	0
Electrocardiogram ST segment depression	0	1 (3.8)	0	0	0	0
Electrocardiogram T wave amplitude increased	0	0	0	1 (2.7)	0	0
Erythema	1 (3.3)	0	0	0	0	0
Gastrointestinal hemorrhage	0	0	0	0	0	1 (5.0)
Gingival infection	0	0	0	1 (2.7)	0	0
Glucose tolerance impaired	0	1 (3.8)	0	0	0	0
Glucose urine present	0	1 (3.8)	0	0	0	0
Gynecomastia	0	1 (3.8)	0	0	0	0
Hypercalcemia	0	0	1 (2.1)	0	0	0

Hyperhidrosis	0	0	0	0	0	1 (5.0)
Hyperkalemia	1 (3.3)	0	0	0	0	0
Hypertriglyceridemia	0	1 (3.8)	0	0	0	0
Insomnia	0	0	0	0	0	1 (5.0)
Loss of consciousness	0	0	0	1 (2.7)	0	0
Malaise	0	0	0	0	0	1 (5.0)
Muscular weakness	0	0	0	0	0	1 (5.0)
Neuropathy peripheral	0	0	0	1 (2.7)	0	0
Neutrophil count decreased	1 (3.3)	0	0	0	0	0
Ocular hyperemia	0	0	0	0	0	1 (5.0)
Palpitations	0	0	0	1 (2.7)	0	0
Papule	1 (3.3)	0	0	0	0	0
Pharyngeal edema	0	0	1 (2.1)	0	0	0
Pleural effusion	0	0	0	0	0	1 (5.0)
Prostatitis	0	1 (3.8)	0	0	0	0
Protein urine	0	0	0	1 (2.7)	0	0
Pruritus	0	0	0	1 (2.7)	0	0
Somnolence	0	0	0	1 (2.7)	0	0
Spinal column stenosis	0	0	0	1 (2.7)	0	0
Stomatitis	0	0	0	1 (2.7)	0	0
Sudden hearing loss	0	0	0	1 (2.7)	0	0
Supraventricular extra systoles	0	0	1 (2.1)	0	0	0
Upper respiratory tract infection	1 (3.3)	0	0	0	0	0
Vertigo positional	0	0	1 (2.1)	0	0	0

TABLE S4 Adverse drug reactions in >2% of patients by ECOG PS (safety analysis set)

	No. of patients (%)			
	ECOG PS 0–1		ECOG PS 2	
	Placebo (*N* = 78)	Anamorelin (*N* = 73)	Placebo (*N* = 12)	Anamorelin (*N* = 10)
Nausea	4 (5.1)	1 (1.4)	1 (8.3)	0
Rash	1 (1.3)	5 (6.8)	0	0
Atrioventricular block first degree	0	4 (5.5)	0	1 (10.0)
Gamma-glutamyltransferase increased	1 (1.3)	3 (4.1)	0	0
Diabetes mellitus	0	2 (2.7)	0	1 (10.0)
Diarrhea	2 (2.6)	0	0	1 (10.0)
Dizziness	2 (2.6)	1 (1.4)	0	0
Glycosylated hemoglobin increased	1 (1.3)	2 (2.7)	0	0
Headache	1 (1.3)	2 (2.7)	0	0
Hyperglycemia	1 (1.3)	2 (2.7)	0	0
Blood creatinine increased	1 (1.3)	0	1 (8.3)	0
Hot flush	0	2 (2.7)	0	0
Hypertension	0	2 (2.7)	0	0
Edema	0	2 (2.7)	0	0
Edema peripheral	0	1 (1.4)	0	1 (10.0)
Pyrexia	0	1 (1.4)	0	1 (10.0)
Tachycardia	0	1 (1.4)	0	1 (10.0)
Vertigo	2 (2.6)	0	0	0
Chest pain	0	0	0	1 (10.0)
Delirium	0	0	0	1 (10.0)
Electrocardiogram QRS complex prolonged	0	0	0	1 (10.0)
Insomnia	0	0	0	1 (10.0)
Malaise	0	0	0	1 (10.0)
Muscular weakness	0	0	0	1 (10.0)

Abbreviations: ECOG PS, Eastern Cooperative Oncology Group Performance Status.
